# Bibliometric analysis of global scientific literature on vaccine hesitancy in peer-reviewed journals (1990–2019)

**DOI:** 10.1186/s12889-020-09368-z

**Published:** 2020-08-17

**Authors:** Waleed M. Sweileh

**Affiliations:** Department of Physiology, Pharmacology/Toxicology, Division of Biomedical Sciences, College of Medicine and Health Sciences, An-Najah National University, Nablus, Palestine

**Keywords:** Vaccine hesitancy, Bibliometric analysis, Influenza vaccines, HPV vaccine

## Abstract

**Background:**

Vaccine hesitancy is a growing threat to national and global health security. The current study was undertaken to provide insights into the global scientific literature on vaccine hesitancy in peer-reviewed journals.

**Method:**

The current study was a descriptive bibliometric study. A validated search strategy on vaccine hesitancy was implemented using SciVerse Scopus. Bibliometric indicators such as (1) annual growth of publications, (2) key players, (3) research themes, (4) pathogens/diseases encountered, (5) top-cited documents, and (6) annual growth of publications stratified by world region and by age category were presented. The study period was from 1990 to 2019.

**Results:**

Search strategy found 2791 documents. The *h*-index of the retrieved literature was 89. The leading journal was *Vaccine* (369; 13.2%%) followed by *Human Vaccines and Immunotherapeutics* (129; 4.6%). Authors from the USA led with 1125 (40.3%) followed by authors from Italy (234; 8.4%) and the UK (204; 7.3%). The US CDC led with 140 (5.0%) documents followed by Emory University (USA) (81, 2.9%). The list of active authors included six from the USA while the remaining were from France, Australia, and Canada. Research themes in the retrieved literature focused on influenza, human papillomavirus, and the role of parents in immunization of their children. The region of the Americas and the European region had the greatest share of publications and showed steep growth of publications lately. Vaccine hesitancy research on adolescents was most noticed after 2007 while that on children was present all the time but escalated lately. Titles/abstracts analysis indicated that 621(22.3%) documents focused on influenza, 392 (14.0%) focused on Human papillomavirus, and 292 (10.5%) focused on measles. The top-cited documents in literature published after 2015 focused on the definition and strategies to overcome vaccine hesitancy.

**Conclusion:**

The last decade witnessed a noticeable increase in the number of publications. Influenza vaccine and parental concerns about the human papillomavirus vaccine were the main focus of the retrieved literature. Information on vaccine hesitancy needs to be collected from all countries to build a better coalition against the anti-vaccination groups. Re-building trust in vaccines requires targeting parents by providing adequate information on the vaccines.

## Background

Vaccination is an important tool to achieve several health targets in Sustainable Development Goals [1, 2]. Despite the scientific and historical evidence of the safety and efficacy of vaccines and vaccination, certain well-organized and well-financed groups are still hesitant and unwilling to accept vaccines and vaccination [3]. Despite that the concept of vaccine hesitancy is complex and challenging [4], the definition proposed by Strategic Advisory Group of Experts (SAGE) Vaccine Hesitancy Working Group of World Health Organization (WHO) [5] is the most commonly reported in the scientific literature. The SAGE stated the following: “vaccine hesitancy refers to delay in acceptance or refusal of vaccines despite availability of vaccine services”. The SAGE definition is sometimes referred to as the ‘3Cs’ model of vaccine hesitancy: confidence, complacency, and convenience [5, 6]. Vaccine-hesitant people exist in the middle of a continuum ranging from full acceptors to total rejecters [6]. The WHO SAGE definition emphasizes that vaccine hesitancy can be specific to a particular vaccine but not others [7]. The Assessment Report of the Global Vaccine Action Plan recommended that each country should develop a strategy to increase acceptance and demand for vaccination by increasing community engagement, trust-building, active hesitancy prevention, regular national assessment of vaccine concerns, and crisis response planning [8]. The growing concern about vaccine hesitancy led the WHO to identify vaccine hesitancy as one of the top ten global health challenges in 2019 [9]. The WHO believes that vaccine hesitancy might hinder its 2019 goals to intensify efforts to prevent cervical cancer using human papillomavirus (HPV) vaccine. Vaccine hesitancy has contributed to the resurgence of poliomyelitis northern Nigeria and measles and pertussis outbreaks in several parts of the world which led to economic loss [10, 11]. The rising problem of vaccine hesitancy was discussed by world leaders at a global health summit [12]. Low trust in vaccination was described as a global crisis and a setback in the fight against deadly, yet preventable infectious diseases and sudden outbreaks [12]. Mistrust in vaccination was augmented post a research paper published in *The Lancet* claiming potential association of vaccination with autism (the paper was retracted 12 years after publication) [13, 14]. The anti-vaccination groups, undoubtedly, have contributed to the increasing sense of fear from vaccination [15]. The impact of the anti-vaccine movement on public health was raised as a question to the European Parliament for investigation [16]. The serious negative role of social media such as Facebook in the spread of misinformation about vaccine and vaccination led Facebook to ban the anti-vaxx ads [17]. Experts believe that high national vaccine coverage rates are needed to maintain herd and community immunity against several infectious diseases [18]. Public health experts also believe that misinformation about vaccines on social media threatens the cumulative achievements in the fight against infectious diseases and that better governmental regulations are needed in this regard [19]. The negative role of media and the impact of counter campaigns in the uptake of the vaccine was shown by a population-based study where HPV vaccine uptake fell significantly below the vaccine uptake baseline in response to negative media coverage and the uptake was recovered upon counter-information campaign [20].

Bibliometrics, an emerging field of information science, is useful for gaining insights into a research activity to identify hotspots and academically significant and landmark publications [21]. Bibliometric analysis helps researchers and funding agencies to focus more on under-investigated areas and make reasonable decisions related to public health. A recent bibliometric study on the top 100 cited articles on vaccines [22] showed that none of the top-cited articles was about vaccine hesitancy suggesting the need for more bibliometric studies to shed light on the research growth and research pattern on vaccine hesitancy. Bibliometric analysis is different from systematic reviews where literature about a specific research question is retrieved from different databases in addition to grey literature and then screened and filtered to a limited number of documents. In bibliometric analysis, only one database is used and grey literature cannot be incorporated in the retrieved data. Few bibliometric studies on vaccines were reported [23–25], but none was carried out on vaccine hesitancy. Therefore, a bibliometric analysis of peer-reviewed literature on vaccine hesitancy was undertaken. The current study comes as a response to global efforts to rebuild public confidence in the vaccine [26]. The goal of the current study was to create a momentum against vaccine hesitancy particularly in world regions with populations at risk of low immunization rates due to low acceptability of vaccines. Such populations need to be targeted by policymakers to minimize their concerns and negative attitudes toward vaccines.

## Methods

### Database

Bibliometric studies use tools to retrieve and analyze literature. In the current study, SciVerse Scopus was used as the bibliometric tool. The choice of Scopus was based on the idea that it is the largest database and provides necessary functions to perform bibliometric analysis [27–29]. Scopus provides basic or advanced search strategy to retrieve the required documents. In the current study, the advanced search strategy was implemented because it offers a wide variety of functions to retrieve documents with a higher level of accuracy.

### Search strategy

The search strategy was developed and validated after trying different scenarios and search strategies. The use of title/abstract search strategy is known to retrieve too many false-positive and false-negative results. It is also known that the title search only will not be comprehensive. Therefore, we developed a search strategy that included title and title/abstract strategy that included all potentially relevant terms and phrases. The terms used in the title search were: hesit* or reluct* or refus* or mistrust or reject or *confidence or distrust or exemption or skepticism or skepticism. The phrases used in the title/abstract search were “vaccin* hesitan*” or “hesitan* to vaccine*” or “vaccin* refusal” or “refusal to vaccine*” or “vaccin* opposition” or “opposit* to vaccin*” or “antivacc* group*” or “anitvax” or antivaccination or “object* to vaccin*” or “resilience to vaccin*” or “debate against vaccin*” or “vaccin* *compliance” or “vaccine* *adherence” or “resist* to vaccin*” or “incomplete vaccin*” or “misinformation about vaccine*” or “vaccin* criticism*” or “delaying vaccin*” or “anxiety from vaccin*” or “criticism to vaccin*” or “barrier* to vaccin*” or “lack of intent to vaccin*” or “poor completion of vaccin*” or “compulsory vaccin*” or “negative perception about vaccin*” or “ negative attitudes” or “engagement in vaccin*” or “choice to vaccin*” or “awareness about vaccin*” or “knowledge about vaccin*” or “behavi* toward vaccin*” or “poor vaccin* uptake” or “vaccin* uptake rate” or “doubts about vaccine*” or “acceptance of vaccine*” or “acceptability of vaccine*” or “contravers* about vaccine*” or “religious exemption” or “fear from vaccin*” or “belief in vaccin*” or “mandatory vaccin*” or “compulsory vaccin*” or “willingness to accept vaccin*” or “parental control of child* vaccin*” or “willingness to vaccinate” or “willingness to accept vaccin*” or “concerns about safety”. Previously published reviews on vaccine hesitancy were used to help in building the search query [7, 30]. Of particular importance is the study by Larson et al. in which a comprehensive approach was used to retrieve relevant documents on vaccine hesitancy using 10 different academic databases and many different languages [7]. The approach used by Larson et al. focused on the use of three vaccine-related terms and 39 terms related to hesitancy. In the current study, more than 40 phrases and terms were used to retrieve the documents with high precision. The approach adopted in the current study was validated for the absence of false-positive and false-negative results. No language restriction was made. Therefore, both English and non-English documents were included. The search strategy was limited to journal articles and the study period was from 1990 to 2019 to represent three decades. It was of no value to make the study period earlier than 1990 because there were a limited number of articles on this topic. The title search yielded 729 documents while the title/abstract search yielded 2518 documents. The combination of both strategies retrieved 2791. Due to the overlap between the two strategies, the sum of the results was not additive (Additional file 1). This number should not be compared with the total number of documents retrieved in systematic reviews of vaccine hesitancy because the initial numbers obtained in systematic reviews included many duplicate documents and false positives and that is why the authors of the systematic reviews have to remove duplicates and false-positive before finalizing the required documents.

### Validation

The validity of the search strategy for minimum false-positive results was confirmed by reviewing the titles and abstracts of the top 100 cited articles obtained by the search strategy. After sharpening the advanced search strategy, the review of the top 100 cited articles found zero false-positive articles. The validity for the minimum false-negative results (missing results) was confirmed by correlating the data obtained for top ten active authors with the data present in Scopus personal profile of the same active authors. The correlation was higher than 95% suggesting a minimum number of missing results. For example, the search strategy retrieved 56 documents authored by Omer S.B., 44 documents by Salmon, D.A, 33 documents by Larson, H. J., on vaccine hesitancy during the study period from 1990 to 2019. Reviewing Scopus profile of Omer, S.B., Salmon, D.A, and Larson, H. J. indicated a similar number of documents on vaccine hesitancy for each of them suggesting a high level of agreement between the number of retrieved documents for active authors and the actual output for the same authors on their Scopus profile. Similar results were obtained for other active authors. This validation method was used in previously published bibliometric studies [31].

### Bibliometric indicators

The current study presented the following bibliometric indicators: volume and growth of publications and citation analysis, top ten active journals, countries, institutions/organization, and authors, types of vaccines by disease/pathogen mostly encountered, visualization of most frequent terms, distribution of documents by world region, distribution of the documents by the age category, and citation analysis including the top ten cited documents. All data were exported from Scopus to Microsoft Excel for data analysis and presentation. For the top ten prolific authors and institutions, the data were calculated based on the number of times the name of the author or institution appeared in the retrieved literature regardless of the position of the author or institution in the author list. For the top ten active countries, the data were calculated based on country affiliation for each author on the article regardless of the position of the author. For example, if an article was published by two authors, one from China and one from the USA, then the article will be counted once for the USA and once from China. An article with authors having the same country affiliation is counted once for that country. This type of calculation is carried out automatically by Scopus without the intervention of the researcher. For the top-cited documents, the study period was divided into two-time intervals and for each time interval, the top five cited documents were presented. The two-time intervals were from 1990 to 2014 and the second one was from 2015 to 2019. The time intervals were created such that the number of documents in each interval was approximately equal. The list of pathogens/diseases investigated was obtained from a previously published bibliometric analysis on vaccines [32]. For citation analysis, the total number of citations and *h*-index were presented [33]. VOSviewer was used to visualize and create maps of most frequently encountered terms in titles/abstracts of the retrieved literature [34]. For graphics, Statistical Package for Social Sciences (IBM SPSS Statistics for Windows, Version 21.0. Armonk, NY: IBM Corp) was used for this purpose. For analysis of research output by geographic region, the WHO classification was used: the region of Americas, African region, the Eastern Mediterranian region, the South Eastern Asian region, the Western Pacific region, and the European region. For analysis of research output based on the age category, the search query was run with specific keywords for each age category. For example, the terms adolescent, adolescence, youth, and high school boys and girls were used to search for literature on adolescents. For adults, the terms adults, woman, man, women, females, and males were used. The same was carried out using child/children for children age category.

### Statistical analysis

The mean number of publications on various age categories and from various world regions were compared using paired samples t-test with a priori *p-*value of 0.05. The SPSS program was used to perform the statistical test.

## Results

### Growth and citation analysis

**Fig. 1:**
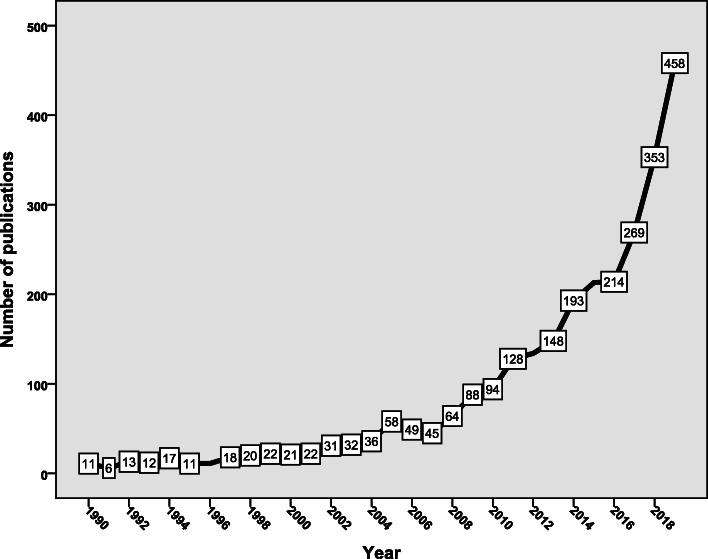
Annual growth of publications on vaccine hesitancy (1990–2019)

The search strategy found 2791 documents, an average of 93 documents per year. More than half (*n* = 1507, 54.0%) of the retrieved documents were published in the last 4 years of the study period (2015–2019) (Fig. 1). Analysis of the types of retrieved documents showed that research articles were the most common type (*n* = 2180; 78.1%) followed by review articles (*n* = 301; 10.8%), notes (*n* = 124; 4.4%); letters (*n* = 78; 2.8%), editorials (*n* = 46; 1.6%), short surveys (*n* = 25; 0.9%), conference papers (*n* = 32; 1.1%) and undefined (*n* = 5, 0.2%). The majority of the retrieved documents (*n* = 2505; 89.8%) were in English while the remaining were in non-English, particularly French, German, Italian, Spanish, and Polish. The retrieved documents received 44,164 citations, an average of 15.8 citations per document. The *h*-index of the retrieved documents was 89. The document that received the highest citation was published in 2007 in *Preventive Medicine*. The document received 571 citations [35].

### Top ten active journals

**Fig. 2:**
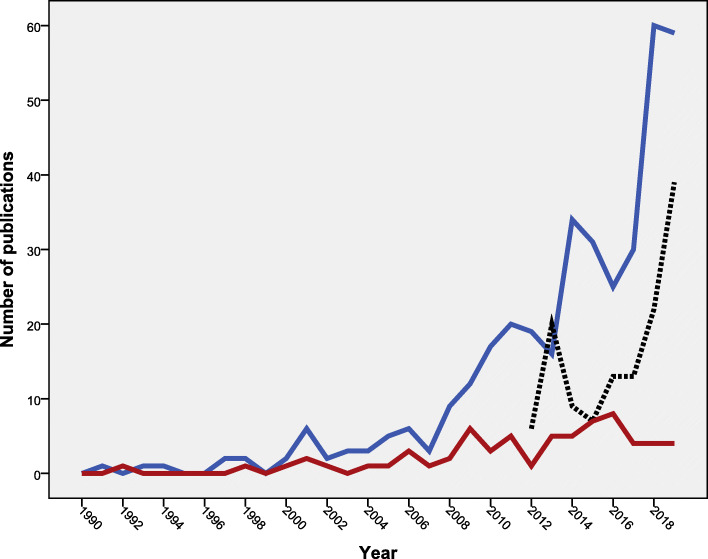
Annual growth of publications on vaccine hesitancy in the top three active journals. Blue line: *Vaccine* journal. Black line: *Human Vaccines and Immunotherapeutics* journal. Red line: *Pediatrics* journal

The leading journal in publishing documents on vaccine hesitancy was *Vaccine* (*n* = 369; 13.2%) followed by *Human Vaccines and Immunotherapeutics* (*n* = 129; 4.6%). Three of the top ten active journals were on vaccines, three on public health, two on infection control, one on pediatrics, and one was multidisciplinary (Table 1). The annual growth of publications of the top three journals showed that the *Vaccine* and *Pediatric* journals published documents on vaccine hesitancy since the 1990s while the *Human Vaccines and Immunotherapeutics* started publishing documents on vaccine hesitancy after 2012 (Fig. 2). The subject areas of the publishing journals were mainly in medicine (*n* = 2407; 86.2%) followed by microbiology/immunology (*n* = 674; 24.1%), and biochemistry/genetics (*n* = 511; 18.3%), with the possibility of overlap between subject areas.

**Table 1:** Top ten active journals in publishing documents on vaccine hesitancy (1990–2019)

Rank^**a**^	Journal	Frequency	% ***N*** = 2791	SJR	Rank	Country
1	*Vaccine*	369	13.2	1.759	Q1	Netherlands
2	*Human Vaccines and Immunotherapeutics*	129	4.6	0.989	Q2	USA
3	*Pediatrics*	66	2.4	2.996	Q1	USA
4	*BMC Public Health*	52	1.9	1.338	Q1	UK
5	*Plos One*	50	1.8	1.1	Q1	USA
6	*American Journal of Public Health*	23	0.8	2.51	Q1	USA
6	*Expert Review of Vaccines*	23	0.8	1.603	Q1	UK
8	*American Journal of Infection Control*	22	0.8	1.07	Q1	USA
9	*Infection Control and Hospital Epidemiology*	21	0.8	1.54	Q1	USA
10	*American Journal of Preventive Medicine*	20	0.7	2.68	Q1	Netherlands

*Q* Quartile, with Q1 being the best in quality and Q4 being the least in quality

*SJR* Scientific Journal Rank. The higher the SJR the stronger the journal

^a^In ranking, two equally active journals were given similar rank and one position in the ranking system was skipped

### Top ten active countries and institutions

Authors from the USA led with 1125 (40.3%) documents followed by authors from Italy (234; 8.4%) and the UK (204; 7.3%). The top ten active countries included two in northern America, six in Europe, two in the Western Pacific region (Table 2). Authors from the top ten active countries participated in 2354 (84.3%) documents.

**Table 2:** Top ten active countries in publishing documents on vaccine hesitancy (1990–2019)

Rank	Country	Frequency	% ***N*** = 2791
1	United States	1125	40.3
2	Italy	234	8.4
3	United Kingdom	204	7.3
4	Canada	182	6.5
5	France	152	5.4
6	Australia	135	4.8
7	Germany	107	3.8
8	Switzerland	88	3.2
9	Netherlands	64	2.3
10	China	63	2.3

The top ten active institutions/ organizations were based mainly in the USA. The US Centers for Disease Control and Prevention (US CDC) led with 140 (5.0%) documents followed by Emory University (USA) (81, 2.9%) and Johns Hopkins University (67, 2.4%) (Table 3).

**Table 3:** Top ten active institutions in publishing documents on vaccine hesitancy (1990–2019)

Rank^**a**^	Institution	Frequency	% ***N*** = 2791	Country Affiliation
1	*Centers for Disease Control and Prevention*	140	5.0	USA
2	*Emory University*	81	2.9	USA
3	*Johns Hopkins University*	67	2.4	USA
4	*University of Washington, Seattle*	52	1.9	USA
4	*London School of Hygiene & Tropical Medicine*	52	1.9	UK
6	*Inserm*	51	1.8	France
7	*The University of Sydney*	50	1.8	Australia
8	*Institut National de Sante Publique Du Québec*	45	1.6	Canada
8	*Aix Marseille Université*	45	1.6	France
8	*Organisation Mondiale de la Santé*	45	1.6	WHO, Geneva
8	*The University of North Carolina at Chapel Hill*	45	1.6	USA

^a^In ranking; two equally active countries were given similar ranks and one position in the rank was skipped

### Research output based on WHO region

**Fig. 3:**
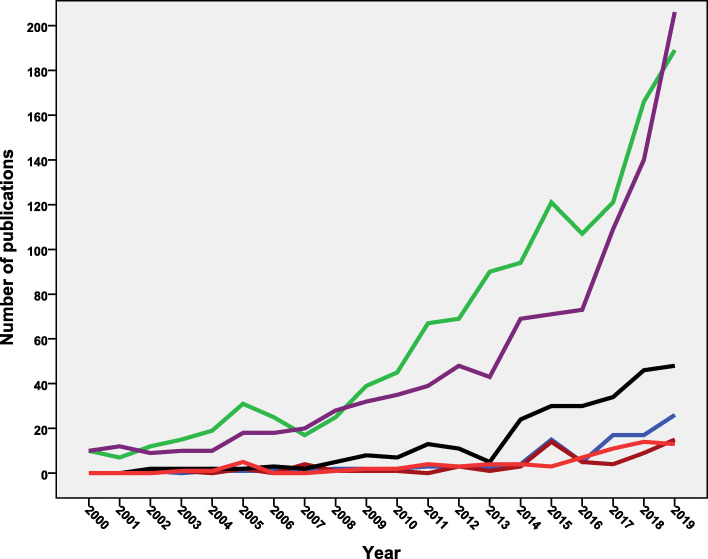
Annual growth of publications on vaccine hesitancy (1990–2019) from the six different WHO world regions. Green line = the region of the Americas; the purple line = the European region; the black line = the Western Pacific region; the blue line = the African region; the light red line = South-East Asian region; and finally the dark red line = the Eastern Mediterranian region

Analysis of research output showed that the region of the Americas (*n* = 1315; 47.1%) ranked first followed by the European region (*n* = 1051; 37.7%) and the Western Pacific region (*n* = 283; 10.1%). The South-East Asian region (*n* = 68; 2.4%) had the least contribution. Figure 3 shows the annual growth of research from the six WHO regions during the period from 2000 to 2019. Both the region of the Americas and the European region showed a steady increase in the number of publications over the study period. The Western Pacific region showed a noticeable contribution to vaccine hesitancy research in the last decade with negligible contribution before 2010. The remaining three world regions showed the least contribution and minimum annual growth with time. The mean number of publication from the region of the Americas was significantly higher (*p* = 0.005) than that from the European region. However, the number of publications from the European region showed a steeper increase in the past 5 years. The European region had significantly (*p* = 0.001) higher mean number of publications from that of the Western Pacific region.

### Top ten active authors

**Fig. 4:**
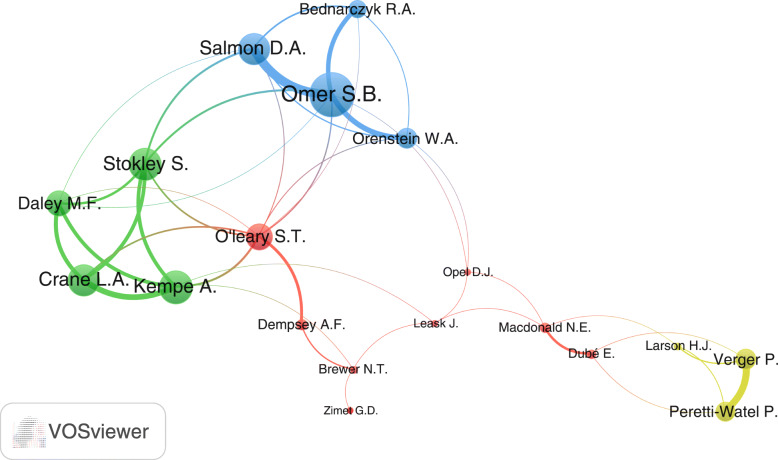
Visualization of research networks of authors with minimum of 15 documents on vaccine hesitancy (1990–2019). Authors in the same cluster had close research interest. The thickness of the connecting line between any two authors represents the strength of research collaboration. Node size reflects the extent of overall research collaboration of the author

Analyses of active authors showed that Omer, S.B. and Salmon D. A. were the leading authors in vaccine hesitancy with 56 (2.0%) and 34 (1.2%) documents respectively. Both authors were from the USA. The top ten active authors (Table 4) included six from the USA, two from Canada, one from France and one from Australia. Visualization of the active authors with at least 15 documents yielded a map with 19 inter-connected researchers and three non-connected researchers (Betsch, C., Signorelli C., and Szucs, T.D). The connected researchers existed in four research networks (clusters). The largest network of researchers (red) included researchers from the USA, Canada, and Australia (Fig. 4).

**Table 4:** Top ten active authors in publishing documents on vaccine hesitancy (2009–2018)

Rank^**a**^	Author	Frequency	% ***N =*** 2791	Affiliation
1	Omer, S.B.	56	2.0	Emory University, Atlanta, United States
2	Salmon, D. A.	44	1.6	Johns Hopkins, Baltimore, United States
3	Larson, H. J	33	1.2	Institute for Health Metrics and Evaluation, Seattle, United States
4	Verger, P.	28	1.0	Observatoire Regional de la Sante Provence-Alpes-Cote d'Azur, Marseille, France
5	MacDonald, N.E.	27	1.0	Dalhousie University, Halifax, Canada
5	Leask, J.	27	1.0	The University of Sydney, Sydney, Australia
7	Dube, E.	22	0.8	Institut National de Sante Publique Du Québec, Quebec, Canada
7	Opel, D.J.	22	0.8	University of Washington, Seattle, Seattle, United States
9	Dempsey, A.F.	21	0.8	University of Colorado at Boulder, Boulder, United States
9	Stokley, S	21	0.8	National Center for Immunization and Respiratory Diseases, United States

^a^In ranking; two equally active countries were given similar ranks and one position in the rank was skipped

### Research themes

**Fig. 5:**
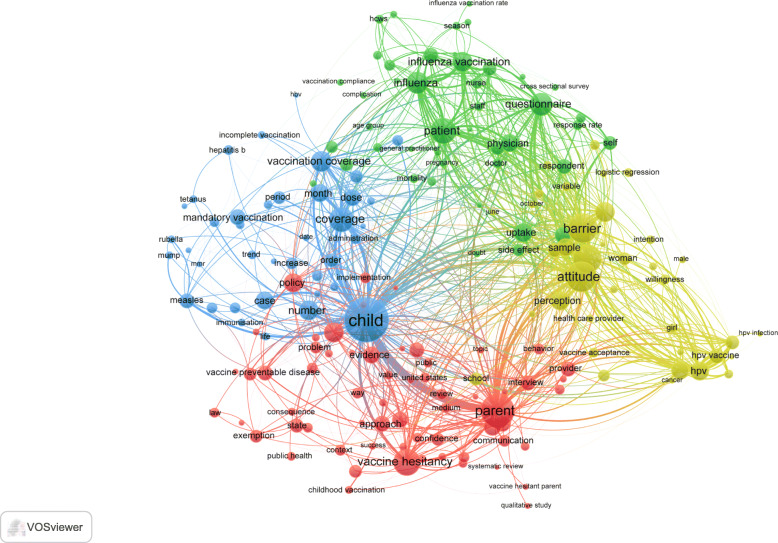
Network visualization map of research themes through mapping of most frequent terms in titles/abstracts of the retrieved documents. A minimum threshold of 50 occurrences was used

Visualization of most frequently encountered terms in title and abstracts of the retrieved documents (minimum of 50 occurrences) yielded four clusters representing four research themes (Fig. 5). The first cluster (red) focused on vaccine hesitancy in general. The second cluster (yellowish-green) focused on the parental concerns, girls, cancer and the HPV vaccine. The third cluster (green) focused on the influenza vaccine/vaccination, and the fourth cluster focused on child vaccination including mumps, MMR (measles, mumps, and rubella), rubella, polio, diphtheria, tetanus, and hepatitis. Retrieved documents focused on different populations of interest. 1266 (45.4%) targeted parents, 662 (23.7%) documents targeted healthcare workers/providers, and 111 (4.0%) targeted pregnant women.

### Common pathogens/diseases encountered in the retrieved literature

**Fig. 6:**
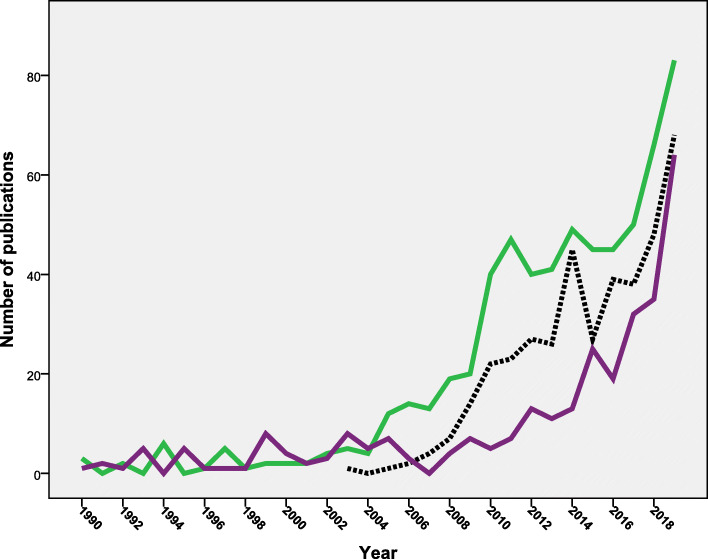
Annual growth of publications on the top three pathogens/diseases encountered in the retrieved literature on vaccine hesitancy (1990–2019). Green line = Influenza. Black line = Human Papillomavirus. Purple line = Measles

The retrieved documents were analyzed for most commonly encountered disease/pathogen. In the titles/abstracts of the retrieved literature. In total, 2811 (100.7%) documents mentioned one or more of the disease/pathogen listed in Table 5. Out of 2791 documents, 621 (22.3%) focused on the influenza, 392 (14%) on the HPV, and 292 (10.5%) on the measles. The annual growth of publications on influenza, HPV, and measles-related vaccine hesitancy showed a steep rise in the last 4 years of the study period (Fig. 6).

**Table 5:** Diseases/pathogens encountered in the title/abstract of the retrieved literature on vaccine hesitancy (1990–2019)

Disease/Pathogen	Frequency	% ***N*** = 2791
Influenza	621	22.3
Human Papillomavirus	392	14.0
Measles	292	10.5
Polio	147	5.3
Pertussis	173	6.2
Hepatitis	250	9.0
Tetanus	147	5.3
Rubella	139	5.0
Diphtheria	150	5.4
Varicella	83	3.0
Rotavirus	44	1.6
Mumps	133	4.8
Pneumococcus	136	4.9
Tuberculosis	26	0.9
Herpes	23	0.8
Smallpox	39	1.4
Rabies	16	0.6
**Total**	**2811**	**100.7**

### Top cited documents

The top five cited documents published before 2015 included articles about HPV and review articles about vaccine hesitancy [7, 35–38]. The top five cited documents published after 2015 discussed issues related to the definition of vaccine hesitancy and strategies needed to overcome the problem [30, 39–41].

### Age category

**Fig. 7:**
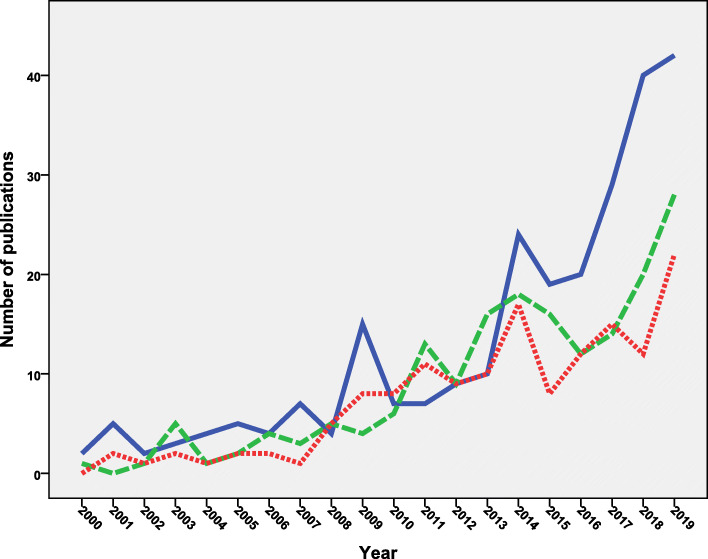
Annual growth of publications on vaccine hesitancy on different age categories. Blue line = children. Green = adults. Red = adolescents

The retrieved documents were also analyzed for age category. When the analysis was made in titles and abstracts, more than one third (*n* = 1021; 36.6%) of the retrieved documents discussed vaccine hesitancy in children, 574 (20.6%) documents discussed vaccine hesitancy among adults, and 475 (17.0%) discussed vaccine hesitancy among adolescents. The remaining documents (*n* = 721; 25.8%) did not specify age category. Figure 7 shows the annual growth of publications for age category mentioned in the “title” of the retrieved documents for the period from 2000 to 2019 to show the difference in growth in a clear way. Research on vaccine hesitancy on adolescents started after 2007 which coincided with the introduction of HPV vaccine. Research on vaccine hesitancy on adults seemed to match the outbreaks of influenza virus. No significant difference was found in the mean number of publications on adults versus adolescents (*p* = 0.07). Research on vaccine hesitancy for children was present all the time but escalated in the last few years due to the aggressive campaigns of anti-vax groups. The mean number of publications on children was significantly higher than that on adults (*p* = 0.003) and adolescents (*p* = 0.001).

### Intervention studies

The retrieved documents were also analyzed for interventional studies to promote vaccine uptake and overcome vaccine hesitancy. The search retrieved 135 interventional studies, more than half of them (*n* = 71; 52.6%) were published from the USA while the remaining were published from Italy, Australia, Canada, South Africa, Switzerland, and others.

## Discussion

The current study aimed to do a bibliometric analysis of peer-reviewed literature on vaccine hesitancy. The current study showed more than a seven-fold increase in the number of publications on vaccine hesitancy during the study period. The serious outbreaks of measles in the USA and other parts of the world were a strong alarming signal to researchers and health policymakers about vaccine hesitancy [42, 43]. For example, during 2019, New York State witnessed an outbreak of over 200 confirmed measles cases attributed to low vaccination rates among certain religious groups [44]. A second potential reason for this upsurge in research on vaccine hesitancy was the huge volume of fake information about vaccines on social media [45] which encouraged several researchers to respond and demonstrate support for vaccines and vaccination [46]. A third potential reason for this upsurge of research was due to a series of safety concerns and concerns among some that the HPV vaccination would make girls promiscuous [47, 48]. The first HPV vaccine became available in 2006 and the uptake of the vaccine is an important step in eliminating cervical cancer globally [49]. The fourth potential reason for the upsurge in the number of publications on vaccine hesitancy was the influenza pandemic. The US Centers for Disease Control and Prevention estimated that 151,700–575,400 people worldwide died from the 2009 H1N1 flu pandemic (swine flu) in the first year of the pandemic [50]. The final potential reason for the increase in the number of publications on vaccine hesitancy is the global work done by the WHO and the Working Group on Vaccine Hesitancy. The Working Group defined two indicators to monitor vaccine hesitancy: Indicator 1: Reasons for vaccine hesitancy and Indicator 2: Percentage of countries that have assessed the level of hesitancy towards vaccination at the national or subnational level in the previous 5 years [5]. These indicators encouraged researchers and health policymakers to find out national data regarding both indicators [51].

The retrieved publications on vaccine hesitancy received a high number of citations indicative of a large number of readers and scholars. For example, the *h*-index of the retrieved literature on vaccine hesitancy was 90 which is higher than that reported for literature on infection-related topics such as strongyloidiasis (*h*-index = 76) [52] and epidermal parasitic skin diseases (*h*-index = 74) [53] but lower than that on carbapenem resistance (*h* index = 102) [54]. The high number of citations for literature on vaccine hesitancy could be attributed to the fact that vaccine hesitancy is of interest to scientists in several fields including public health, pediatrics, microbiology/immunology, psychology, social sciences, and others. Another potential reason for high citations is the leading role of international health organizations on vaccine hesitancy. Both the WHO and the US CDC had an active role in this topic and had specialized groups to investigate the subject [55, 56]. A third potential reason is the reputation of journals publishing the retrieved documents. Most of the top ten active journals publishing documents on vaccine hesitancy were ranked as Q1 in their specific fields and had a relatively high Scientific Journal Ranking (SJR). A fourth potential reason is the seriousness of the topic. In 2019, WHO listed vaccine hesitancy as one of the top ten global health threats [9]. According to the WHO, immunization prevents 2–3 million deaths a year globally and that 1.5 million more deaths can be prevented yearly if the vaccination rates reach the target level [55]. A fifth potential reason is the presence of a good percentage of documents on vaccine hesitancy as review articles. Six out of the top twenty cited documents were review articles and almost 11% of the retrieved documents were review articles [57].

The current study indicated that the top ten active journals in publishing documents on vaccine hesitancy were prestigious and specialized journals with high SJR. The presence of three vaccine-related journals in the top list was justifiable. These three specialized journals need to lead the fight and to uncover the truth about vaccines using evidence-based medicine and publishing certain thematic supplements on this subject to minimize harm made by the anti-vaccination groups. The same argument applies to public health experts and physicians who should participate in eliminating parental vaccine hesitancy by focusing on the seriousness of the infection rather than on the vaccine itself. A study found that motivational interviewing helped improve HPV vaccine series initiation by 9.5% among parents who were resistant to vaccinating their teens [58]. This partially explains the presence of pediatric journals as well as public health journals in the active list. A second potential reason for the presence of pediatric and public health journals in the active list was the active role of primary healthcare pediatric physicians in educating and confronting myths about vaccines.

The current study indicated that more than half of the retrieved documents included authors from Northern America and Europe. Furthermore, of the top ten active countries, none was in Latin America or the Middle East or South-Eastern Asian region. This was unsurprising due to several reasons. Researchers in the USA and Europe ranked first in various scientific disciplines [53, 59]. Second, the anti-vaccination movement was originally initiated in the UK and gained momentum in the USA for religious and non-religious reasons [60]. Third, the fake scientific evidence linking vaccination with autism also started in the USA and the UK which increased the participation of researchers in these countries to get more involved in this debate. Fourth, the idea that democratic countries with a wide margin of freedom of speech are more prone to vaccine hesitancy. However, high research output from developed countries should not be misinterpreted. Vaccine hesitancy is an old and worldwide problem that varies from one country to another. A study compared vaccine hesitancy in five low- and middle-income countries (Bangladesh, China, Ethiopia, Guatemala, and India) using the WHO’s 10-item Vaccine Hesitancy Scale [61]. The study found that mothers in Bangladesh perceived less vaccination benefit compared with mothers in China. However, mothers in Ethiopia and Guatemala perceived greater benefit than mothers in China and that educational level was not significantly linked with vaccine hesitancy. The Wellcome Global Monitor Report (2018) indicated that countries, where people are most likely to agree that vaccines are safe, included Bangladesh, Egypt, Ethiopia, Liberia, Tanzania, and India. The report stated that “ only 72% of people in Northern America,73% in Northern Europe59% in Western Europe, 50.0% in Eastern Europe, 95% in South Asia and 92% in Eastern Africa agree that vaccines are safe. In France, one in three people disagrees that vaccines are safe, the highest percentage for any country worldwide. The highest percentage of parents who said their children did not receive a vaccine were Southern Africa, 9% and East Asia and Southeast Asia, 8%” [62]. Based on this argument, the low research output from certain world regions should be handled seriously and information regarding indicators 1 and 2 of the Working Group should be rigorously searched and presented to health policymakers. It should be emphasized that the reasons for vaccine hesitancy vary by country income level, by WHO region and over time and within a country [51].

The current study showed that the African region, the Middle Eastern region, and the South-Eastern Asia region had the least contribution to the literature on vaccine hesitancy. Vaccination programs in Africa had made tremendous success in eliminating several dangerous infectious diseases [63]. However, the success made in Africa is threatened by the rise of vaccine hesitancy in certain parts in Africa. A striking example is the boycott of the polio vaccine in Nigeria in 2003–2004 driven by rumours and distrust [63]. The problem of vaccine hesitancy is also in the rise in the eastern Mediterranian region and urgent action is needed [64]. The Eastern Mediterranean Vaccine Action Plan 2016–2020 published by the WHO recommended enforcing legislation that should cover the vaccine hesitancy/refusal, especially regarding vaccination of adolescents and adult age groups, as well as the implementation of supplementary immunization activities for measles-containing vaccines [64]. Similar concerns were raised about vaccine hesitancy in Southeast Asian region given the rise of certain preventable infectious diseases such as measles [65].

The current study indicated that literature on vaccine hesitancy focused mainly on the influenza vaccine, HPV vaccine, and to a lesser extent on the measles vaccine. It is estimated that 3–5 million new cases of influenza are registered each year causing a lot of morbidity and mortality in children, elderly, and severely ill patients [66]. The 2009–2010 H1N1 influenza pandemic uncovered the low rates of influenza vaccine uptake, which led to global serious health consequences [67]. The 2009–2010 H1N1 influenza pandemic explains the rise in the number of publications seen in Fig. 2 during the 2010–2011 period. A systematic review of the potential barriers for influenza vaccination suggested that low worry, low perceived risk of the disease, doubts about the safety and effectiveness of the vaccine, lack of trust in health authorities, and knowledge gaps were the main barriers for influenza vaccination [68]. In young children, parental hesitancy to vaccinate their children against influenza has been attributed to concerns about vaccine safety [69–71]. Healthcare workers are key players in discussing vaccine hesitancy with parents and the public [72]. Studies have shown that interventions and personal communications are needed to increase awareness, knowledge, understanding, and uptake of influenza vaccine among healthcare workers who represent a trustful source for the public about the vaccine [73, 74]. In the current study, the number of publications on influenza vaccine hesitancy was the highest for reasons related to the optional and seasonal nature of influenza in addition to the wrong belief among the public that influenza vaccine causes influenza [75]. To overcome influenza vaccine hesitancy, several strategies at all level need to be adopted. To develop such strategies, studies on influenza vaccine hesitancy need to be carried out at country level to pinpoint pockets of vaccine resistance and reasons behind such resistance at the country and group levels.

Results of the current study indicated that the number of publications on the HPV vaccine was second to those on influenza vaccine hesitancy. Human papillomavirus vaccine has made progress in fighting the HPV infections and HPV-associated cervical cancer. The FDA has approved three vaccines for HPV: the bivalent vaccine, the quadrivalent vaccine, and 9-valent vaccine [76, 77]. The introduction of the 9-valent vaccine was not associated with an overall change in HPV vaccination [78]. The nine-valent HPV vaccine was licensed in 2014 for girls and boys age 11 years or older [79]. Association between HPV and cervical cancer is well established and that is why the US CDC recommends that 11–13-year-old girls get vaccinated against HPV [80, 81]. Despite its effectiveness and importance in eliminating potential cancers, the uptake of the HPV vaccine in several countries around the world was low [47]. Studies on HPV vaccine hesitancy showed a lot of public mistrust in the safety of the vaccine as well as on the health system [48]. The focus should be directed toward healthcare workers who have concerns about HPV vaccine safety [82]. It has been reported that more than half a million new cases of cervical cancer are diagnosed annually with high mortality rates registered in poor countries [83, 84].

The current study showed that childhood vaccines were a major category discussed followed by adults and adolescents. The emergence of vaccine hesitancy among adolescents emerged mainly after the development and introduction of the HPV vaccine and that is why the annual growth of publications on vaccine hesitancy among adolescents showed a steep increase after 2007. The same argument applies to vaccine hesitancy among adults which emerged due to the annual influenza vaccination and certain flu outbreaks after 2010. In fact, a major setback of vaccination programs and the emergence of vaccine hesitancy was the fake data linking certain types of vaccination with autism in children.

The current study indicated that most documents on vaccine hesitancy focused on parents. Studies have reported several reasons for parents’ refusal to get their children vaccinated. Such reasons include serious concerns about the safety of vaccines, fear from autism, objection to many injections, religious grounds, and lack of adequate information [85]. To overcome parental vaccine hesitancy, religious and social leaders need to get involved to encourage parents to get their children fully immunized. Furthermore, national and international health bodies need to give primary healthcare providers technical and scientific training to be able to answer and address parental concerns scientifically and rationally.

The current study is different from certain previously published studies on vaccine hesitancy using the systematic review approach. The most related study is the one published by Larson et al. in 2014 under the title “Understanding vaccine hesitancy around vaccines and vaccination from a global perspective: A systematic review of published literature, 2007–2012” [7]. The study retrieved a total of 1164 documents from 10 academic databases with several different languages including Arabic, Chinese, Russian, Spanish, English, French, and others. The Larson et al. study showed that during the study period from 2007 to 2012, publications on adults and adolescents were almost parallel and increasing while that on children was steady. This is similar to the finding in the current study if we focus our attention for the period from 2007 to 2012. However, it is different than the findings of the current study if we focus on a large time scale, for example from 2000 to 2019, simply because the time window for the current study was larger than that used in the study by Larson et al. The finding of the current study regarding the distribution of the retrieved documents on geographical regions was similar to that produced by Larson et al. The current study showed that 47, 37, and 10% were the contribution of the regions of the Americas, European, and Western Pacific respectively. The finding of Larson et al. for these three regions was 46, 27, and 12%. The European region had made a major contribution to global research on vaccine hesitancy while the contribution of the regions of the Americas and Western Pacific region remained approximately the same.

The current study showed that there were 135 interventional studies to increase vaccine uptake and overcome vaccine hesitancy. It is not enough to screen for vaccine hesitancy and to understand the causes of this phenomena. Researchers and policymakers should develop interventional educational materials and videos as well as other techniques to overcome vaccine hesitancy. A systematic review studies on strategies for addressing vaccine hesitancy concluded that multicomponent and dialogue-based interventions were most effective but the strategies should be carefully tailored according to the target population, their reasons for hesitancy, and the specific context [30].

Authors of the current study did their best to provide accurate data analysis. However, the chance of error in the analysis remains a possibility. For example, in listing active authors or institutions, the authors relied on data present in Scopus. Different name spelling or different abbreviations of the names of authors or institutions might create an error in the top ten list. Furthermore, the authors did their best to retrieve all documents on Vaccine hesitancy and tried to validate their search strategy using methods adopted in previously published bibliometric studies. However, there is no perfect search strategy and the potential for false positive or false negative results remains a possibility. Finally, Scopus is the largest database, but it is biased toward English language and journals based in the modern world. Therefore, publications on vaccine hesitancy in unindexed journals in Asia, Africa, the Middle East, and Eastern Europe have been missed.

## Conclusion

The current study is meant to create a positive momentum among researchers all over the world to investigate this topic at the national and group levels. Such information is needed to better organize campaigns on all fronts about the health importance of vaccinations. Both parents and healthcare providers need to be targeted with adequate information about vaccination. HPV vaccine hesitancy is on the rise as indicated by the number of publications. World regions with a high prevalence of HPV-related cancers need to organize campaigns to young females and males as well as parents to shed light on the danger of HPV infection and the safety and efficacy of the vaccine. Research on influenza vaccine acceptance should be encouraged.

## Supplementary information

**Additional file 1.** Search strategy and keywords used to retrieve documents on vaccine hesitancy.

## Data Availability

all data presented in this manuscript are available on Scopus database using the search query listed in the methodology section.

## Abbreviations

WHO: World Health Organization
CDC: Centers for Disease Control and Prevention
HPV: Human Papillomavirus
SJR: Scientific Journal Rank
Q1: First Quartile
