# Supplementary material for: Bibliometric analysis of global scientific literature on vaccine hesitancy in peer-reviewed journals (1990–2019)
Source: BMC Public Health. 2020 Aug 17;20:1252. doi: 10.1186/s12889-020-09368-z (PMC7433157; doi:10.1186/s12889-020-09368-z)
Supplement: Supplementary file 1 — Additional file 1. Search strategy and keywords used to retrieve documents on vaccine hesitancy. [file 12889_2020_9368_MOESM1_ESM.docx]

**Bibliometric analysis of peer-reviewed literature on vaccine hesitancy (2009 – 2019)**

**Additional file 1**: Search strategy and keywords used to retrieve documents on vaccine hesitancy

| **TITLE** | **Operator** | **TITLE** | **Operator** | **Title/Abstract** |
| --- | --- | --- | --- | --- |
| vaccine or vaccination or immunization or immunisation | and | hesit* or reluct* or refus* or mistrust or reject or *confidence or distrust or exemption or skepticism or skepticism | - | - |
| vaccine or vaccination or immunization or immunisation |  |  | **and** | "parental control of child* vaccin*" or "willingness to vaccinate" or "willingness to accept vaccin*" or "concerns about safety" or "vaccin* hesitan*" or "hesitan* to vaccine*" or "vaccin* refusal" or "refusal to vaccine*" or "vaccin* opposition" or "opposit* to vaccin*" or "antivacc* group*" or "anitvax" or antivaccination or "object* to vaccin*" or "resilience to vaccin*" or "debate against vaccin*" or "vaccin* *compliance" or "vaccine* *adherence" or "resist* to vaccin*" or "incomplete vaccin*" or "misinformation about vaccine*" or "vaccin* criticism*" or "delaying vaccin*" or "anxiety from vaccin*" or "criticism to vaccin*" or "barrier* to vaccin*" or "lack of intent to vaccin*" or "poor completion of vaccin*" or "compulsory vaccin*" or "negative perception about vaccin*" or " negative attitudes" or "engagement in vaccin*" or "choice to vaccin*" or "awareness about vaccin*" or "knowledge about vaccin*" or "behavi* toward vaccin*" or "poor vaccin* uptake" or "vaccin* uptake rate" or "doubts about vaccine*" or "acceptance of vaccine*" or "acceptability of vaccine*" or "contravers* about vaccine*" or "religious exemption" or "fear from vaccin*" or "belief in vaccin*" or "mandatory vaccin*" or "compulsory vaccin*" or "willingness to accept vaccin*" |

Quotation marks were used when the exact phrase is to be retrieved while the asterisk (*) was used as a wild card.

Study period was limited from 1990 to 2019. Retrieved documents were limited to journal documents only. Veterinary documents and irrelevant documents were excluded
